# *Pasteurella multocida* Toxin Triggers RANKL-Independent Osteoclastogenesis

**DOI:** 10.3389/fimmu.2017.00185

**Published:** 2017-02-27

**Authors:** Sushmita Chakraborty, Bianca Kloos, Ulrike Harre, Georg Schett, Katharina F. Kubatzky

**Affiliations:** ^1^Zentrum für Infektiologie, Medizinische Mikrobiologie und Hygiene, Universitätsklinikum Heidelberg, Heidelberg, Germany; ^2^Department of Internal Medicine 3, Institute of Clinical Immunology, University of Erlangen-Nuremberg, Erlangen, Germany

**Keywords:** bacterial toxin, osteoclast, immune evasion, pro-inflammatory cytokines, G protein, *Pasteurella multocida* toxin

## Abstract

Bone remodeling is a continuous process to retain the structural integrity and function of the skeleton. A tight coupling is maintained between osteoclast-mediated resorption of old or damaged bones and osteoblast-mediated formation of new bones for bone homeostasis. While osteoblasts differentiate from mesenchymal stem cells, osteoclasts are hematopoietic in origin and derived from myeloid precursor cells. Osteoclast differentiation is driven by two cytokines, cytokine receptor activator of NF-κB ligand (RANKL), and macrophage colony-stimulating factor. Imbalances in the activity of osteoblasts and osteoclasts result in the development of bone disorders. Bacterially caused porcine atrophic rhinitis is characterized by a loss of nasal ventral conche bones and a distortion of the snout. While *Bordetella bronchiseptica* strains cause mild and reversible symptoms, infection of pigs with toxigenic *Pasteurella multocida* strains causes a severe and irreversible decay. The responsible virulence factor *Pasteurella multocida* toxin (PMT) contains a deamidase activity in its catalytical domain that constitutively activates specific heterotrimeric G proteins to induce downstream signaling cascades. While osteoblasts are inhibited by the toxin, osteoclasts are activated, thus skewing bone remodeling toward excessive bone degradation. Still, the mechanism by which PMT interferes with bone homeostasis, and the reason for this unusual target tissue is not yet well understood. Here, we show that PMT has the potential to differentiate bone marrow-derived macrophages into functional osteoclasts. This toxin-mediated differentiation process is independent of RANKL, a cytokine believed to be indispensable for triggering osteoclastogenesis, as addition of osteoprotegerin to PMT-treated macrophages does not show any effect on PMT-induced osteoclast formation. Although RANKL is not a prerequisite, toxin-primed macrophages show enhanced responsiveness to low concentrations of RANKL, suggesting that the PMT-generated microenvironment offers conditions where low concentrations of RANKL lead to an increase in the number of osteoclasts resulting in increased resorption. PMT-mediated release of the osteoclastogenic cytokines such as IL-6 and TNF-α, but not IL-1, supports the differentiation process. Although the production of cytokines and the subsequent activation of signaling cascades are necessary for PMT-mediated differentiation into osteoclasts, they are not sufficient and PMT-induced activation of G protein signaling is essential for efficient osteoclastogenesis.

## Introduction

Bone is a dynamic tissue, which is constantly remodeled to regulate its structural integrity and functions. Bone remodeling is a physiological process to maintain homeostasis and involves the removal of old or damaged bone structures by osteoclasts and the subsequent replacement of new bone by osteoblasts. A tight coupling of bone resorption to bone formation is maintained in normal bone homeostasis. However, bacterial infections can derail this process and result in bone diseases like caries, periodontitis, osteomyelitis, septic arthritis, lyme disease, and atrophic rhinitis (AR) (1). Bacteria can mediate bone damage by (a) releasing substances, which can directly destroy the bone matrix, (b) by activating osteoclasts or inhibiting osteoblasts through their surface or secreted components, (c) by stimulating inflammatory cells, which subsequently activate osteoclasts resulting in enhanced bone resorption, and (d) by invading osteoblasts resulting in inhibition of bone matrix formation and dysregulation of bone remodeling (2).

One of the most potent bacterial toxin involved in bone destruction is *Pasteurella multocida* toxin (PMT), which is produced by capsular type D or some type A strains of *Pasteurella multocida* (3). PMT is the causative agent of porcine AR, an economically important disease, characterized by degeneration of nasal turbinate bones, leading to a shortening or twisting of the snout (4). In humans, few and rare cases of septic arthritis have been reported due to *P. multocida* infection, and these are usually caused by animal bites (5, 6). PMT is a 146 kDa protein and acts as a potent mitogen for cell types such as osteoclasts or fibroblasts (7, 8). In piglets, administration of purified PMT on nasal conche is sufficient to cause AR (9). The toxin mediates AR through interfering with bone biogenesis by promoting osteoclast differentiation and proliferation resulting in increased bone resorption, while inhibiting osteoblast differentiation and bone regeneration on the other hand (10–14). The mode of action of PMT on host cells is through activation of heterotrimeric G protein families Gα_q/11_, Gα_i1–3_, and Gα_12/13_ but not through Gα_s_ activation (15). PMT induces the permanent activation of heterotrimeric G proteins of the host by deamidation of a conserved glutamine residue in the switch II region of the α subunit, critical to maintain the intrinsic GTPase activity of G protein (16). As a consequence of constitutive activation of host G proteins by PMT, signaling cascades such Map kinase, JAK-STAT, or PI3 kinase pathway are activated, which result in mitogenesis, increased survival, and cytoskeletal reorganization (17). We hypothesize that bone destruction is a side-effect of toxin-induced production of cytokines in an attempt to modulate signal transduction pathways of innate and adaptive immune cells to avoid immune recognition (18).

Osteoclasts differentiate from hematopoietic precursors of the monocyte/macrophage lineage through the action of two cytokines, RANKL and macrophage colony-stimulating factor (M-CSF). Upon stimulation of their cognate receptors, a cascade of signaling events is initiated leading to the activation of transcription factors such as nuclear factor of activated T cells, calcineurin dependent 1 (NFATc1), NF-kB, and AP-1, resulting in the fusion of precursor cells and expression of genes for osteoclast functions (19). Mice deficient in the cytokines M-CSF and RANKL or their cognate receptors display a prominent osteopetrotic phenotype. Osteoprotegerin (OPG) is a soluble receptor that competes with RANK for RANKL and protects the skeleton from excessive bone resorption. Overexpression or administration of OPG in mice results in profound osteopetrosis by reducing osteoclastogenesis (20). Under physiological conditions the RANKL/OPG ratio is balanced to maintain skeletal integrity.

Osteoclastogenic plasticity has been observed across the myeloid lineage ranging from early myeloid precursors to monocytes, macrophages, and dendritic cells, which can differentiate into tartrate-resistant acid phosphatase (TRAP)-positive osteoclasts in the presence of soluble RANKL (sRANKL) and M-CSF (21–23). Studies from our group and others have shown the osteoclastogenic property of PMT in cell lines or heterogeneous precursor populations (24–26); therefore, we decided to investigate the potential of PMT in inducing osteoclastogenesis in a homogeneous population of bone marrow-derived macrophages (BMDMs) to unravel the mechanism of osteoclast differentiation in more detail. We show that PMT drives the differentiation of BMDMs into TRAP-positive cells independent of RANKL-RANK signaling, as treatment of PMT-stimulated BMDMs with OPG did not abrogate osteoclast formation. In addition, our investigation shows that PMT-induced osteoclastogenesis is modulated by cytokines and their downstream signaling pathways that are necessary but not sufficient for efficient differentiation of macrophages into osteoclasts.

## Materials and Methods

### Ethics Statement

All animal studies were approved by the Regierungspräsidium Karlsruhe, Germany.

### Mice

C57BL/6 wild-type mice were purchased from Janvier Labs (Le Genest St. Isle, France), and IL-1R-deficient mice were obtained from Jackson Laboratories (Bar Harbor, ME, USA). Mice were maintained under SPF conditions in accordance with the German policies on animal welfare.

### Reagents

Tissue culture reagents were purchased from Biochrom GmbH (Berlin, Germany), PAA laboratories, PAN biotech (Aidenbach, Germany), Merck and Sigma, respectively. Antibodies against phosphorylated c-Jun (p-c-Jun) (Ser63), p-NF-kB (Ser536), phosphorylated STAT-3 (Tyr705), HistonH3, and β-actin were purchased from Cell Signaling Technology (Frankfurt, Germany). Antibodies against NFATc1 and Gq were procured from Santa Cruz Biotechnology (Heidelberg, Germany). An antibody that recognizes the Q209E modification of Gα_q_ was a gift of Prof. S. Kamitami (Osaka, Japan). Secondary HRP-linked antibodies were obtained from Cell Signaling Technology (anti-rabbit IgG, anti-Mouse IgG) or Santa Cruz (anti-rat IgG). FITC-conjugated anti-CD11b, FITC-conjugated anti-CD80, FITC-conjugated anti-CD40 were from BD Biosciences (Heidelberg, Germany). PE-conjugated anti-CD86 and PECy7-conjugated anti-MHCII were purchased from eBioscience (Frankfurt, Germany). PECy7-conjugated anti-F4/80 and PE-conjugated anti-RANK and the corresponding isotype control were purchased from BioLegend (San Diego, CA, USA).

Rat anti-mouse Interleukin 6 receptor (IL-6R) antibody was a gift from Chugai Pharmaceutical, Tokyo, Japan. Etanercept (Enbrel; Pfizer) used in blocking experiment of TNF-α was generously provided by Prof. G. Schett (Erlangen, Germany) and Prof. H. Lorenz (Heidelberg, Germany). The Anti-IL-6 antibody for blocking IL-6 signaling was obtained from BioXCell (West Lebanon, USA).

PCR primers were purchased from Apara (Denzlingen, Germany) or Biomol (Hamburg, Germany). Recombinant PMT and the catalytically inactive mutant PMT^C1165S^ were kindly provided by Prof. Klaus Aktories (Freiburg).

### Differentiation of BMDMs

Bone marrow (BM) cells were isolated from the femur and tibia of 6–12 weeks old C57BL/6 mice. These cells were then used to generate BMDMs, using L929-cell conditioned medium (LCCM) as a source of granulocyte/M-CSF. On day 1, BM cells were resuspended in 20 ml of complete medium (DMEM supplemented with 10% FBS, 100 U/ml penicillin, 100 µg/ml streptomycin, and 50 µM 2-mercaptoethanol). On day 2, non-adherent cells were collected by flushing the petri dish several times. These cells were then resuspended in complete medium containing 30% LCCM. On day 4, 30% LCCM was again added and incubated for an additional 3 days. To obtain the BMDM, the supernatants were discarded and the attached cells were collected in 10 ml of complete medium and centrifuged at 200 *g* for 5 min.

### Stimulation

Cells were stimulated with 1 nM PMT or 20–100 ng/ml rec. mouse sRANKL, 25 ng/ml rec. mouse M-CSF 100 ng/ml OPG (all from R&D Systems, Abington, UK). For cytokine experiments, cells were treated with 700 pg/ml TNF-α (eBioscience), 2,500 pg/ml IL-6, and 900 pg/ml IL-1β, respectively (Miltenyi, Bergisch-Gladbach, Germany).

### Quantitative Real-time PCR

A total of 1 × 10^6^ BMDM cells were seeded per well in a 6-well plate and then stimulated as indicated. RNA was extracted using High-Pure RNA isolation kit (Roche), according to the manufacturers’ protocol. cDNA was prepared by using Revert Aid First strand cDNA synthesis kit (Thermo Scientific). Quantitative RT-PCR was performed using SYBR Green Rox mix (Thermo Scientific) with the primers listed in Table 1. RT-PCR was performed using the 7900 HT Fast Real-Time PCR System (AB Applied Biosystems). An initial denaturation step of 10 min at 95°C was common for all genes, but the following cycle for annealing and amplification was different. For *Nfatc1, Il1a, Acp5, Ocstamp* (40 cycles at 95°C for 15 s and at 58°C for 1 min); *calcr* (50 cycles at 95°C for 10 s and at 58°C for 45 s); *Tnf, Il6*, and *Il1b* (40 cycles at 95°C for 15 s and at 60°C for 1 min); *Ctsk, Atp6vod2, Oscar* (40 cycles at 95°C for 15 s, 60°C for 30 s, and 72°C for 30 s). As normalization control *Rsp29* was used, relative expression (rE) was calculated as rE = 1/(2^ΔCt^).

**Table 1:** **Primer sequences**.

Gene	Forward	Reverse
*Acp5*	5′-TTC CAG GAG ACC TTT GAG GA-3′	5′-GGT AGT AAG GGC TGG GGA AG-3′
*Oscar*	5′-AGG GAA ACC TCA TCC GTT TG-3′	5′-GAG CCG GAA ATA AGG CAC AG-3′
*Ctsk*	5′-AGG GAA GCA AGC ACT GGA TA-3′	5′-GCT GGC TGG AAT CAC ATC TT-3′
*Nfatc1*	5′-GGG TCA GTG TGA CCG AGG AT-3′	5′-GGA AGT CAG AAG TGG GTG GA-3′
*Ocstamp*	5′-TGG GCC TCC ATA TGA CCT CGA GTA G-3′	5′-TCA AAG GCT TGT AAA TTG GAG GAG T-3′
*Atp6v0d2*	5′-TCA GAT CTC TTC AAG GCT GTG CTG-3′	5′-GTG CCA AAT GAG TTC AGA GTG ATG-3′
*Rps29*	5′-AGC CGA CTC GTT CCT TTC TC-3′	5′-CGT ATT TGC GGA TCA GAC C-3′
*Tnf*	5′-AGC CCC CAG TCT GTA TCC TT-3′	5′-CTC CCT TTG CAG AAC TCA GG-3′
*Il6*	5′-CCG GAG AG GAGA CTT CAC AG-3′	5′-TTC TGC AAG TGC ATC ATC GT-3′
*Il1b*	5′-ACT CAT TGT GGC TGT GGA GAA G-3′	5′-GCC GTC TTT CAT TAC ACA GGA-3′
*Il1a*	5′-CGG GTG ACA GTA TCA GCA AC-3′	5′-GAC AAA CTT CTG CCT GAC GA-3′
*calcr*	5′-AGA GTG AAA AGG CGG AAT CT-3′	5′-TTT GTA CTG AGC ATC CAG CA-3′
*Tnfrsf11a*	5′-CGA GGA AGA TTC CCA CAG AG-3′	5′-CAG TGA AGT CAC AGC CCT CA-3′

### ELISA

Supernatants of stimulated cells were harvested and analyzed for IL-6 and TNF-α using mouse IL-6 ELISA MAXTM Standard Set and mouse Tnf-α ELISA MAXTM Standard Set, respectively (BioLegend, San Diego, CA, USA). A TecanGENios Pro plate reader (Tecan, Crailsheim, Deutschland) was used for quantification. Results were analyzed using the Magellan5 software.

### Determination of Bone Resorption Pit Area

A total of 2.5 × 10^5^ cells were plated in 1 ml of complete medium in 24-well plates and treated as mentioned. On day 3, cells were transferred to a 96-well plate containing bovine cortical bone slices (http://Boneslices.com, Jelling, Denmark) and cultivated for 15–21 days. For measurement of resorbed bone, bone slices were washed with phosphate buffer saline (PBS), incubated in 5% sodium hypochlorite for 1–2 h, washed thoroughly with water, and stained with 0.1% toluidine blue. The pits developed a blue to purple color. The resorbed area was calculated from micro images with Adobe^®^ Photoshop^®^ CS5.

### TRAP Staining

A total of 2.5 × 10^5^ cells were plated in 1 ml of complete medium in 24-well plates and treated as described in figure legends. Cells were then fixed and stained using Acid Phosphatase, Leukocyte (TRAP) Kit (Sigma, St. Louis, MO, USA). TRAP-positive cells with three or more nuclei were scored as osteoclasts.

### Western Blot Analysis

A total of 1 × 10^6^ cells were stimulated in 2 ml of complete medium in a 6-well format as indicated with PMT. For the lysis, cells were washed twice with ice-cold PBS and collected in ice-cold PBS by scrapping. Cell pellets were lysed in 200 µl of 1× NP40 buffer, freshly supplemented with a Phosphatase and Protease-Inhibitor Cocktail (Roche). Lysates were separated by SDS-PAGE (4–20% gradient polyacrylamid gel, Anamed). Proteins were transferred to nitrocellulose membrane *via* semi-dry western blot, blocked in TBST (5% BSA) for 1 h at RT before the membranes were incubated with the primary antibody, diluted as suggested by Cell Signaling Technology over night at 4°C. After 1 h incubation with the secondary antibody (HRP-coupled), protein bands were detected by enhanced chemiluminescence.

### Nuclear Extract Preparation

A total of 1.2 × 10^7^ BMDMs were stimulated as indicated in the figure legend in 10 ml of complete medium. Nuclear extracts were prepared using a nuclear extraction kit (Active Motif). Briefly cells were washed and then collected by scrapping in ice-cold PBS containing phosphatase inhibitor. Cells were then centrifuged at 500 *g* for 10 min at 4°C. Pellet was resuspended in 500 µl of 1× hypotonic buffer and incubated for 15 min on ice. Then 25 µl of detergent was added and vortexed for 10 s. Cells were then centrifuged at 14,000 *g* for 1 min at 4°C. Supernatant containing the cytoplasmic fraction was transferred into new microcentrifuge tubes. The pellet was washed with PBS twice, resuspended in 50 µl of complete lysis buffer, vortexed, and incubated for 30 min rocking on ice. After that the samples were centrifuged at 14,000 *g* for 10 min, and the supernatant containing the nuclear fraction was collected. Western Blot analysis was performed with the nuclear fraction.

### Cathepsin K Activity Assay

A total of 5 × 10^5^ BMDMs were seeded per well in a six-well plate. Cells were then stimulated with either PMT or M-CSF or M-CSF+sRANKL for 6 days, and cathepsin K Activity Assay was performed (Abcam). Cells were lysed in 200 µl of cathepsin K cell lysis buffer and incubated on ice for 10 min. Cell debris were centrifuged at 14,000 *g* for 5 min, and the supernatants were removed. The amount of protein in the lysates was determined with a BCA Assay (Pierce), and the amount was adjusted to 3 µg of protein in 50 µl of lysis buffer per well of a 96-well plate. Fifty microliters of cathepsin K reaction buffer was added to each well. The Ac-LR-AFC substrate was added to a final concentration of 140 µM, and the plate was incubated for 2 h at 37°C. Fluorescence was measured with a microplate reader (FLUOstar OPTIMA; BMG LABTECH) with an excitation of 355 nm and an emission of 520 nm.

### FACS Analysis

For FACS analysis 1 × 10^6^ cells were used per sample. Cells were blocked for 15 min in PBS, 2% BSA on ice in a total volume of 100 µl before staining the cells for 1 h on ice with the appropriate antibody or incubated in the corresponding isotype control diluted as suggested by manufacturers. Surface expression of RANK (R12-31), CD11b (M1/70), CD80 (16-10A1), CD86 (GL-1), F4/80 (BM8), CD40 (3/23), and MHCII (M5/114.15.2) was quantified by flow cytometry on a FACSCanto cytometer (BD Biosciences, Heidelberg, Germany). For RANK expression, the mean fluorescence intensity was recorded and the values were corrected for differences in basal fluorescence of unstained cells. Overlays were generated using Flowing Software 2.5.

### Phagocytosis Assay

5 × 10^5^ cells were incubated in 100 µl of complete medium at 37°C or at 4°C with green fluorescent latex beads (diameter: 1 µm) diluted 1:100 for 1 h. Afterward, cells were washed five times with ice-cold PBS, and phagocytosis was measured by performing flow cytometry (FACSCanto cytometer, BD Biosciences, Heidelberg, Germany).

### Cytokine and Inhibitor Experiments

A total of 2.5 × 10^5^ cells were plated in 1 ml of complete medium in 24-well plates and stimulated according as detailed in the figure legends. For the analysis of cytokine-mediated effects, cytokines were added to the appropriate stimuli or were added alone as cytokine mix directly afterward. Half of the medium was changed after 1½ days cytokines were replenished. After 10–12 days, TRAP staining was performed. To address the role of IL-1, Tnf-α and IL-6 signaling in PMT-induced osteoclastogenesis, BMDMs were generated from wt-mice and IL1-R-deficient mice and were treated with the inhibitor etanercept (Enbrel; Pfizer) with a concentration of 120 µg/ml and a neutralization antibody for murine IL-6 (anti m IL-6, BioXCell) at 105 µg/ml for 1 h prior to stimulation. Cells were stimulated with PMT and M-CSF, and the inhibitors were again added 12 h after stimulation. Forty-eight hours after addition of the inhibitors, half of the medium was changed and stimulated or inhibited, respectively. After 10–12 days, a TRAP stain was performed.

### Statistical Analyses

Data are presented as means ± SD. Comparison between the groups was performed by employing a Student’s *t*-test. *p* values ≤0.05 were considered statistically significant. Multiple-group comparisons were analyzed by analysis of variance.

## Results

### PMT Triggers Osteoclast Formation in Mouse BMDMs

**Figure 1:**
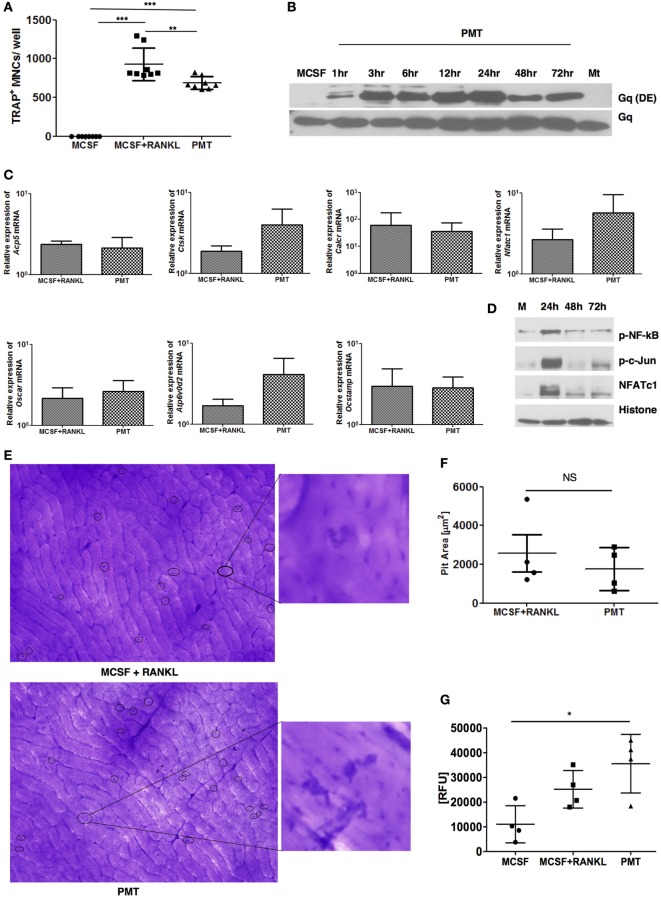
***Pasteurella multocida* toxin (PMT) induces differentiation of bone marrow-derived macrophages (BMDMs) into osteoclasts**. **(A)** BMDMs were stimulated with standard concentrations of macrophage colony-stimulating factor (M-CSF), M-CSF/sRANKL, and PMT for 6–10 days as described in Section “Materials and Methods.” Cells were then fixed and stained for tartrate-resistant acid phosphatase (TRAP) activity and TRAP^+^ multinucleated cells (MNCs) were counted. The indicated SD was obtained from four experiments (mean ± SD; *n* = 4). Statistical analysis was performed using analysis of variance (ANOVA) followed by Bonferroni’s multiple comparison test (***p ≤ 0.0005; **p ≤ 0.005). **(B)** To investigate the kinetics of PMT-mediated Gα_q_ deamidation, cells were stimulated for the indicated time points with 1 nM PMT prior to lysis. As a control, cells were either stimulated with M-CSF or a catalytically inactive mutant of PMT (PMT^C1165S^, Mt) for the longest time point. The immunoblot was probed with an antibody detecting the deamidated form of Gα_q_ (Q209E) or total Gα_q_ (*n* = 3). **(C)** Quantitative RT-PCR analysis of gene expression of *Acp5* (TRAP), *Ctsk* (cathepsin K), calcitonin receptor (*Calcr*), *Nfatc1, Oscar, Atp6v0d2*, and *Ocstamp* in BMDMs treated either with M-CSF, M-CSF/sRANKL, or PMT; the results were normalized to *Rps29* expression. Cells were stimulated for 12 h to check the expression of *Nfatc1* and *Ocstamp*; for 24 h to check the expression of *Oscar*; for 48 h to check the expression of *Acp5, Ctsk*, and *Atp6v0d2*; and for 72 h to check the expression of *Calcr*. The data are presented as fold change relative to the expression of M-CSF-treated cells at the same time point. The indicated SD was obtained from three or more experiments (mean ± SD; *n* ≥ 3). No significant difference was observed comparing gene expression of PMT-treated samples with M-CSF/sRANKL-treated sample using a paired Student’s *t*-test. **(D)** Immunoblot of phosphorylated NF-κB (p-NF-κB), phosphorylated c-Jun (p-c-Jun), NFATc1, and histone in nuclear extracts from BMDM treated either with PMT or M-CSF (M) for the indicated time points; histone was used as a loading control (*n* = 3). **(E)** Representative photographs of resorption pits (left panel) and magnified image of a resorption pit (right panel) induced by M-CSF + RANKL or PMT. Resorption pit is marked with circle. **(F)** The diagram represents the pit area that was calculated by deducting the pit area value of M-CSF + RANKL or PMT, respectively, from MCSF-treated wells (*n* = 4). Resorption pit pictures were evaluated in a blinded fashion, and false-positive pits were excluded by marking similar structures in M-CSF-treated samples. No significant difference was observed between PMT-treated samples with M-CSF/sRANKL-treated samples using a paired Student’s *t*-test (ns, not significant). **(G)** Cells were stimulated with M-CSF, M-CSF/sRANKL, and PMT and lysed as described in Section “Materials and Methods.” Cathepsin K activity was analyzed by fluorescence detection measured in duplicates (*n* = 4). Statistical analysis was performed using one-way ANOVA comparing cells stimulated with M-CSF/sRANKL and PMT to the M-CSF sample (*p ≤ 0.05).

*Pasteurella multocida* toxin was shown to have the potential to differentiate osteoclast precursors into osteoclasts in pig, mouse, and rat models using heterogeneous and mostly ill-defined precursor populations (11, 25, 27). As none of these studies checked the osteoclastogenic potential of PMT on a homogenous population of macrophages devoid of stromal or osteoblastic contamination, we decided to investigate the effect of PMT in BMDMs as a model system. After an initial characterization of the population for the expression of typical macrophage markers that allow to distinguish macrophages from monocytes (Figure S1 in Supplementary Material) (28), macrophages were washed and stimulated with PMT, in the absence of M-CSF or other cytokines. PMT treatment resulted in TRAP-positive osteoclast formation with PMT comparable to stimulation with M-CSF/sRANKL (Figure 1A; Figure S2 in Supplementary Material). We then tested whether the observed osteoclastogenesis in BMDMs was due to a direct effect of PMT. As PMT is known to deamidate the alpha-subunit of heterotrimeric Gq, we checked the kinetics of deamidation of Gq in BMDMs (29). We observed a very rapid uptake of the toxin resulting in the deamidation of Gq already after 1 h of PMT stimulation that persisted for at least 72 h (Figure 1B). We next compared the expression of osteoclast markers in PMT-treated macrophages along with M-CSF/sRANKL-treated macrophages. Real-time qPCR analysis revealed that PMT stimulation in macrophages induced expression of NFATc1 (*Nfatc1*), acid phosphatase 5, tartrate-resistant (*Acp5*, TRAP), cathepsin K (*Ctsk*), d2 isoform of vacuolar (H+) ATPase (v-ATPase) V0 domain (*Atp6v0d2*, ATP6v0d2), osteoclast associated receptor (*Oscar*, OSCAR), calcitonin receptor, and osteoclast stimulatory transmembrane protein (*Ocstamp*, OC-STAMP) (Figure 1C). We further checked the activation of transcription factors essential for driving osteoclastogenesis and observed an activation of NFATc1, c-Jun, and NF-kB after PMT stimulation (Figure 1D). Next, we sought to examine whether PMT-induced osteoclasts were able to resorb cortical bovine slices. Indeed, PMT-stimulated osteoclasts derived from pure macrophages were able to efficiently resorb bone matrix (Figures 1E,F). Further characterization revealed significant cathepsin K activity in PMT-treated cells, suggesting that PMT induces differentiation of macrophages into functional osteoclasts (Figure 1G). Together, these observations suggest that PMT induces direct differentiation of BMDMs into functional osteoclasts.

### PMT Induces Differentiation of Macrophages into Osteoclasts in a RANKL-Independent Manner

Osteoprotegerin is a soluble secreted protein of the TNF receptor superfamily that is also known as osteoclast inhibitor factor (30). Both OPG and RANK are receptors for RANKL. OPG is an antagonistic endogenous receptor that inhibits osteoclastogenesis. The OPG–RANKL complex counterbalances the effect of the RANK–RANKL complex, thus playing an important role in maintaining bone homeostasis.

**Figure 2:**
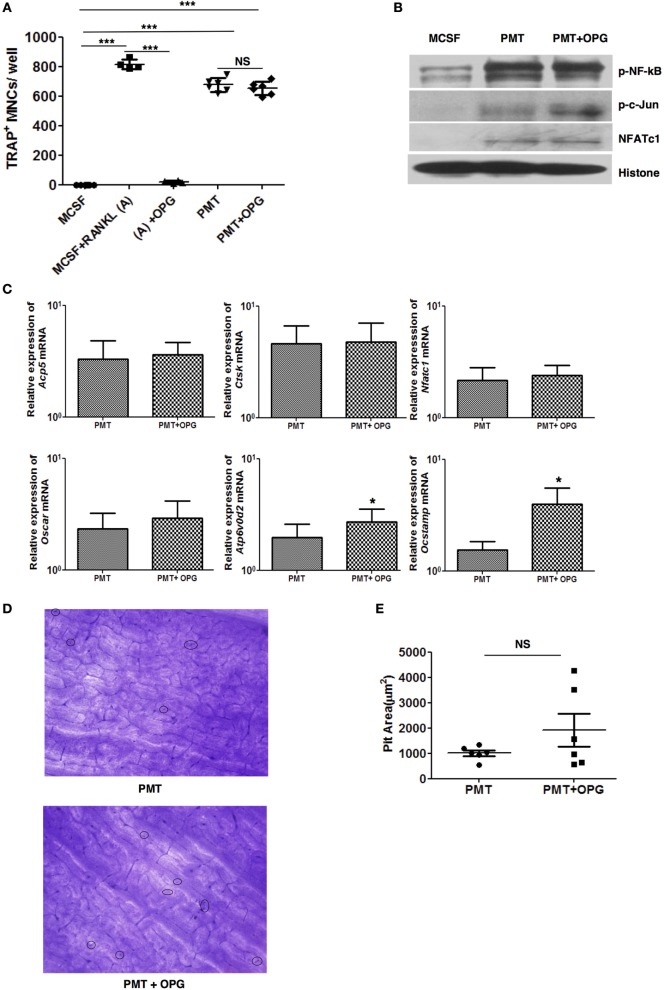
***Pasteurella multocida* toxin (PMT)-mediated osteoclastogenesis is unaltered with osteoprotegerin (OPG) treatment**. **(A)** Bone marrow-derived macrophages (BMDMs) were stimulated either with PMT, PMT/OPG, M-CSF/sRANKL, M-CSF/sRANKL/OPG, or macrophage colony-stimulating factor (M-CSF) alone for 6–10 days (PMT 1 nM; OPG 100 ng/ml; M-CSF 25 ng/ml; sRANKL 100 ng/ml). Multinucleated TRAP^+^ cells were counted per well. The indicated SD was obtained from two or more experiments measured in duplicates (mean ± SD; *n* ≥ 2). Statistical analysis was performed using ANOVA followed by Bonferroni’s multiple comparison test (***p ≤ 0.0005; NS, not significant). **(B)** BMDMs were treated with M-CSF, PMT, or with PMT/OPG for 24 h, and nuclear extracts were prepared. The immunoblot shows phosphorylated NF-κB (p-NF-κB), phosphorylated c-Jun (p-c-Jun), NFATc1, and histone, which was used as a loading control (*n* = 2). **(C)** Quantitative RT-PCR analysis of gene expression of *Acp5, Ctsk, Ocstamp, Nfatc1, Oscar*, and *Atp6v0d2* in BMDMs treated either with M-CSF, PMT/OPG, or PMT; values were normalized to *Rps29* expression. Data are presented fold change relative to the gene expression of M-CSF-treated cells at the same time point. Cells were stimulated for 12 h to check the expression of *Nfatc1* and *Ocstamp*; for 24 h to check the expression of *Oscar*; and for 48 h to check the expression of *Acp5, Ctsk*, and *Atp6v0d2*. The indicated SD was obtained from three or more experiments (mean ± SD; *n* ≥ 3). Statistical analysis was performed using a paired Student’s *t*-test comparing gene expression of PMT/OPG-treated cells to the PMT-treated cells (*p ≤ 0.05). **(D)** Representative picture of bone resorption assays performed with PMT-stimulated cells with or without OPG. Resorption pits are marked with circles. **(E)** The histogram represents the calculated pit area that was obtained by subtracting the pit area value of PMT or PMT/OPG wells from M-CSF-treated conditions (*n* ≥ 3). The resorption pit pictures were evaluated in a blinded fashion and the false-positive pits were excluded by marking similar structures in M-CSF-treated samples. Statistical analysis was performed using a paired Student’s *t*-test comparing PMT + OPG treatment with PMT-treated sample (NS, not significant).

As PMT induces differentiation of macrophages into osteoclasts, we wanted to check the effect of OPG on PMT-induced osteoclastogenesis. We treated macrophages with OPG along with M-CSF/sRANKL or PMT, respectively, and then assessed osteoclast formation. Concomitant treatment with OPG completely abrogated osteoclast formation after M-CSF/sRANKL stimulation but failed to show any significant effect on PMT-mediated osteoclast formation (Figure 2A). To exclude that PMT might outcompete OPG at the concentration used (1 nM), we gradually decreased PMT concentrations to as low as 0.01 nM, but no variation in the number of TRAP-positive osteoclasts was found (Figure S3 in Supplementary Material). Furthermore, we checked the effect of OPG on PMT-induced signaling pathways and transcription factors. In the presence of OPG, PMT was still able to activate NFATc1, NF-kB, and c-Jun (Figure 2B). We validated this observation by checking the expression of osteoclast markers induced by PMT in the presence of OPG (Figure 2C). OPG treatment of macrophages along with PMT did not alter the expression of *Nfatc1, Acp5, Oscar*, and *Ctsk* but upregulated the expression of *Ocstamp* and *Atp6v0d2* compared to PMT-treated macrophages. Next, we checked the bone resorptive potential of osteoclasts derived from PMT treated with OPG. Again, we did not observe any loss of function of PMT-generated osteoclasts in the presence of OPG, suggesting that OPG treatment does not interfere with PMT-mediated osteoclastogenesis (Figures 2D,E). Together, these data prove that PMT-induced differentiation of macrophages into osteoclasts is RANKL-RANK signaling independent.

### PMT Primes Macrophages for Enhanced Response to RANKL Stimulation

**Figure 3:**
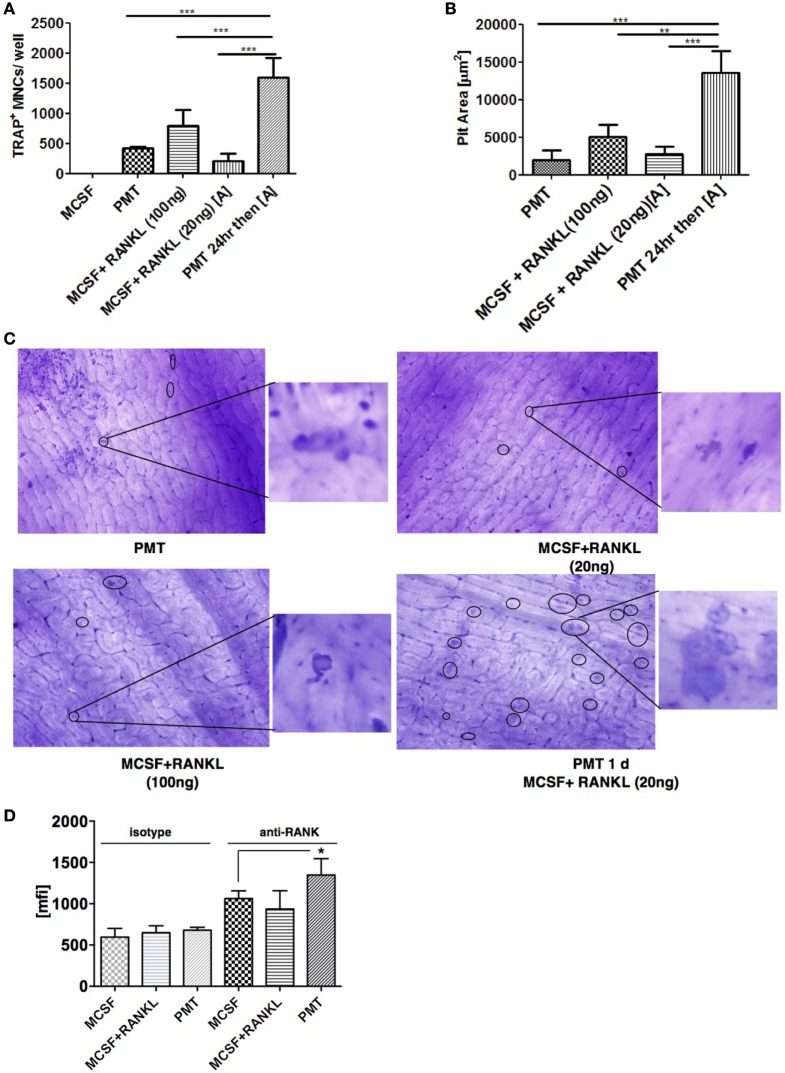
***Pasteurella multocida* toxin (PMT) pre-treatment augments M-CSF/RANKL-induced osteoclastogenesis in macrophages**. **(A)** Bone marrow-derived macrophages were treated with stimuli as indicated for 10 days using high (100 ng/ml) and low (20 ng/ml) concentrations of RANKL. TRAP^+^ multinucleated cells (MNCs) were counted (mean ± SD; *n* = 2). Statistical analysis was performed using analysis of variance (ANOVA) followed by Bonferroni’s multiple comparison test comparing TRAP^+^ cells treated with PMT for 1 day before switching to low M-CSF/sRANKL concentrations with samples treated only with PMT, M-CSF/sRANKL (high), or M-CSF/sRANKL (low), respectively (***p ≤ 0.0005). **(B)** Graph of the calculated pit area that was obtained by deducting the pit area value of macrophage colony-stimulating factor (M-CSF)-treated conditions from the respective samples (mean ± SD; *n* = 3). Resorption pit pictures were evaluated in a blinded fashion, and false-positive pits were excluded by marking similar structures in M-CSF-treated samples. Statistical analysis was performed using ANOVA followed by Bonferroni’s multiple comparison test comparing pit areas in samples treated with PMT for 1 day before switching to M-CSF/sRANKL (low) with PMT, M-CSF/sRANKL (high), or M-CSF/sRANKL (low) samples (**p ≤ 0.005; ***p ≤ 0.0005). **(C)** Representative photographs of resorption pits (left panel) and magnified image of a resorption pit (right panel). Resorption pit is marked with circle. **(D)** FACS analysis of RANK surface expression after 1 day of treatment with M-CSF/sRANKL or PMT (mean ± SD; *n* = 3). Statistical analysis of RANK expression was performed using ANOVA followed by Bonferroni’s multiple comparison test (*p ≤ 0.05).

As PMT-mediated osteoclast formation was not blocked by the RANKL inhibitor OPG, we wanted to see if PMT could influence RANKL-mediated signaling pathways. To check if PMT might augment the response of macrophages at low concentrations of RANKL, we stimulated macrophages with PMT for 1 day, then carefully washed the macrophages with culture medium and re-stimulated them with a low amount of sRANKL (20 ng) in the presence of M-CSF. We observed a marked increase in TRAP-positive cells when the cells had been pretreated with PMT compared to the treatment with sRANKL/M-CSF or PMT, alone (Figure 3A). This resulted in an enhanced number of resorption pits (Figures 3B,C). These observations demonstrate that PMT increases the responsiveness of the stimulated progenitor cells for RANKL-mediated osteoclastogenesis.

We next determined whether PMT modulates the expression of RANK in order to increase the responsiveness of RANKL. We observed a slight increase in surface expression but not gene expression of RANK at 24 h of treatment with PMT compared to M-CSF-treated samples (Figure 3D; Figures S4A,B in Supplementary Material). However, we wondered, if there were other effects involved in the increased activity of cells pretreated with PMT, given the strong effect of the PMT pretreatment on pit formation. As we had seen before that PMT is a strong inducer of pro-inflammatory genes, we investigated whether these cytokines influence the process of differentiation induced by PMT in macrophages.

### PMT-Induced TNF-Alpha and IL-6 Are Important Modulators of Its Osteoclastogenic Potential

**Figure 4:**
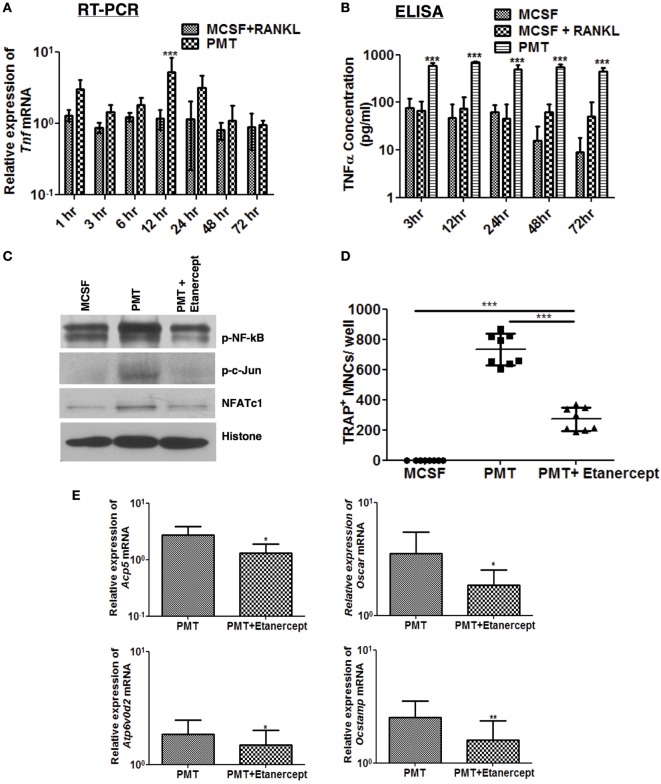
**TNF-α modulates *Pasteurella multocida* toxin (PMT)-induced osteoclastogenesis**. **(A,B)** Bone marrow-derived macrophages (BMDMs) were stimulated with standard concentration of macrophage colony-stimulating factor (M-CSF), M-CSF/sRANKL, or PMT for the indicated the time points. **(A)** Quantitative RT-PCR analysis of *Tnf* normalized to *Rps29* expression. Data are presented fold change relative to the expression of M-CSF-treated cells at the same time point (mean ± SD; *n* = 3). **(B)** ELISA of Tnf-α production (mean ± SD; *n* = 3). Statistical analysis was done by two-way analysis of variance (ANOVA) (***p ≤ 0.001). **(C)** BMDMs were stimulated with PMT, PMT/etanercept (120 µg/ml) or M-CSF for 24 h, and nuclear extracts were prepared. Immunoblots of nuclear phosphorylated NF-κB (p-NF-κB), c-Jun (p-c-Jun), NFATc1, and histone were performed (*n* = 2). **(D)** BMDMs were stimulated with PMT, PMT/etanercept (120 µg/ml), or M-CSF for 10 days. Multinucleated TRAP^+^ cells were counted per well. The indicated SD was obtained from four experiments (mean ± SD; *n* = 4). Statistical analysis was performed using ANOVA followed by Bonferroni’s multiple comparison test comparing TRAP^+^ cells in PMT/etanercept-treated wells to the PMT- and M-CSF-treated wells; TRAP^+^ cells in PMT-treated wells to the M-CSF-treated wells (***p ≤ 0.0005). **(E)** Quantitative RT-PCR analysis of gene expression of *Acp5, Oscar, Atp6v0d2*, and *Ocstamp* in BMDMs treated either with PMT, PMT/etanercept, or M-CSF; data were normalized to *Rps29* expression. Cells were stimulated for 12 h to check the expression of *Ocstamp*; for 24 h to check the expression of *Oscar*; and for 48 h to check the expression of *Acp5* and *Atp6v0d2*. The data are presented as fold change relative to the expression of M-CSF-treated cells at the same time point. The indicated SD was obtained from three or more experiments (mean ± SD; *n* ≥ 3). Statistical analysis was performed using a paired Student’s *t*-test comparing gene expression of PMT/etanercept-treated cells to the PMT-treated cells (*p ≤ 0.05; **p ≤ 0.005).

We have previously shown that PMT is a strong inducer of NF-kB, subsequently causing the induction of pro-inflammatory genes (18). This inflammatory reaction occurs independently of TLR4 or the inflammasome through G-protein-mediated RhoA signaling (31). Therefore, we investigated whether pro-inflammatory cytokines influence PMT-mediated osteoclast differentiation. Tnf-α is secreted by various cell types, including macrophages, and is one of the most potent osteoclastogenic cytokines produced during inflammation. It plays an important role in the pathogenesis of rheumatoid arthritis and other forms of chronic inflammatory osteolysis (32, 33). Therefore, we investigated the kinetics of expression and secretion of Tnf-α after PMT stimulation in macrophages. We observed that PMT induces the expression of Tnf-α starting after 1 h of stimulation, with a maximum induction after 12 h of stimulation (Figure 4A) and continuous secretion of Tnf-α for at least 72 h (Figure 4B).

We thus questioned, whether blocking of Tnf-α signaling by addition of etanercept, a Tnf-α antibody, affects PMT-induced downstream signaling pathways. Addition of etanercept prior to PMT stimulation in macrophages abrogated p-c-Jun and decreased NFATc1 and NF-kB levels in nuclear extracts (Figure 4C). The number of TRAP-positive cells was significantly reduced (approximately 63%) (Figure 4D), and osteoclast-specific gene expression was impaired (Figure 4E). Collectively, these results suggest that PMT-induced Tnf-α production increases its osteoclastogenic potential.

**Figure 5:**
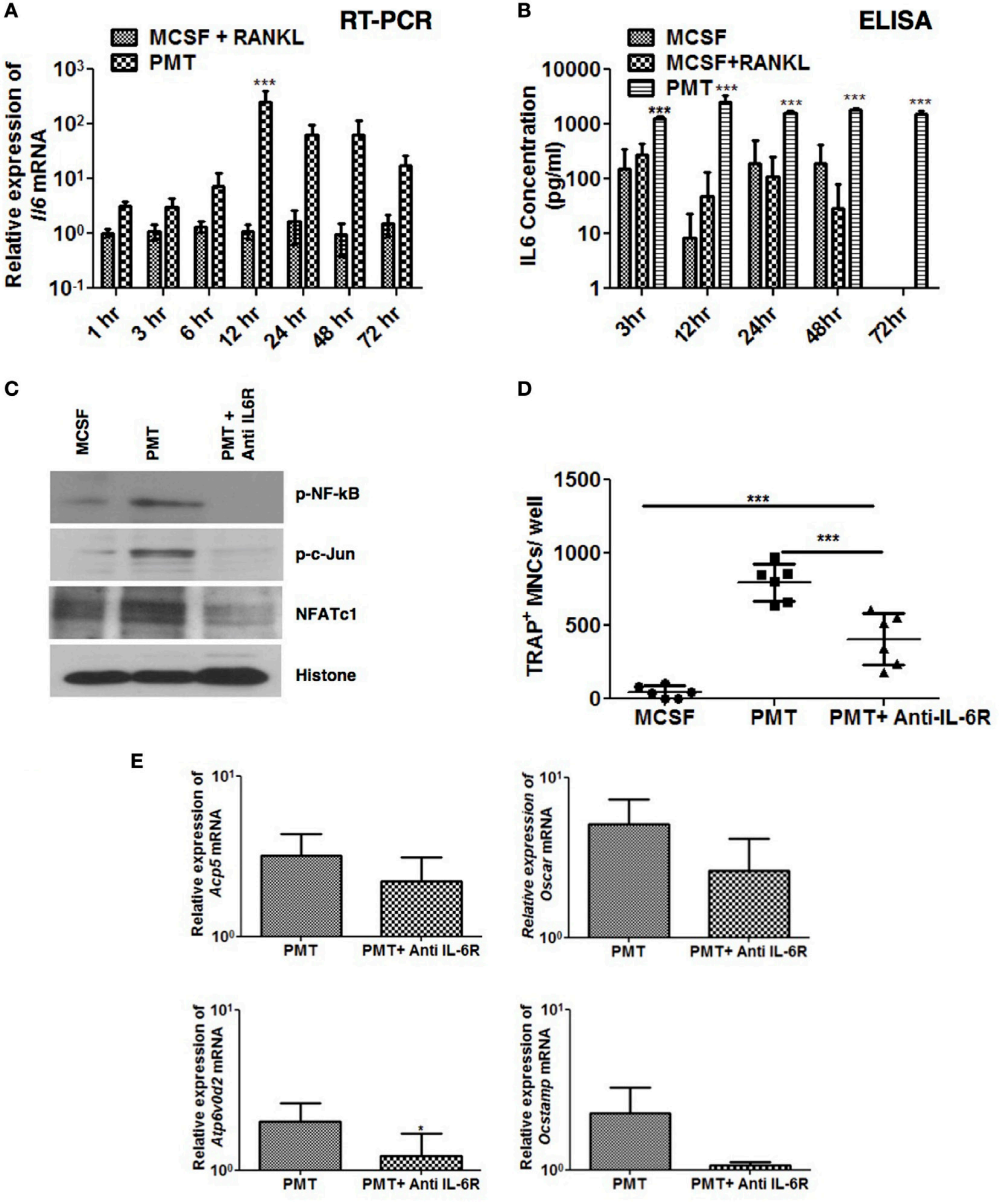
***Pasteurella multocida* toxin (PMT)-mediated osteoclastogenesis is inhibited with blockade of Interleukin 6 receptor (IL-6R). (A,B)** Bone marrow-derived macrophages (BMDMs) were stimulated with standard concentrations of macrophage colony-stimulating factor (M-CSF), M-CSF/sRANKL, or PMT for the indicated time points. **(A)** Quantitative RT-PCR analysis of *Il6* mRNA; normalized to *Rps29* expression. Data are presented fold change relative to the expression of M-CSF-treated cells at the same time point (mean ± SD; *n* = 3). **(B)** ELISA of IL-6 production (mean ± SD; *n* = 3). Statistical analysis was done by analysis of variance (ANOVA) (***p ≤ 0.001). **(C)** BMDMs were stimulated with PMT, PMT/anti IL-6 R (105 µg/ml), or M-CSF for 24 h, and nuclear extracts were prepared. A representative immunoblot of the phosphorylated forms of NF-κB (p-NF-κB) and c-Jun (p-c-Jun), as well as NFATc1 and histone are shown; histone was used as a loading control (*n* = 2). **(D)** BMDMs were stimulated with PMT, PMT/Anti IL-6R (105 µg/ml), or M-CSF for 10 days. Multinucleated TRAP^+^ cells were counted per well. The indicated SD was obtained from three experiments (mean ± SD; *n* ≥ 3). Statistical analysis was performed using ANOVA followed by Bonferroni’s multiple comparison test comparing TRAP^+^ cells in PMT/Anti IL-6R-treated wells to the PMT- and M-CSF-treated wells; TRAP^+^ cells in PMT-treated wells to the M-CSF-treated wells (***p ≤ 0.0005). Statistical analysis was performed using a paired Student’s *t*-test comparing (***p ≤ 0.0005). **(E)** Quantitative RT-PCR analysis of gene expression of *Acp5, Oscar, Atp6v0d2*, and *Ocstamp* in BMDMs treated either with PMT, PMT/Anti-Il6 R (105 µg/ml), or M-CSF alone; values were normalized to *Rps29* expression (mean ± SD; *n* = 3). Cells were stimulated for 12 h to check the expression of *Ocstamp*; for 24 h to check the expression of *Oscar*; and for 48 h to check the expression of *Acp5* and *Atp6v0d2*. The data are presented as fold change relative to the expression of M-CSF-treated cells at the same time point. Statistical analysis was performed using a paired Student’s *t*-test comparing gene expression of PMT/Anti IL-6R-treated cells with PMT-treated cells (*p ≤ 0.05).

In a previous study, we showed that PMT strongly induces interleukin-6 expression of immune cells (34). In mice, overexpression of IL-6 leads to arthritis while absence of IL-6 prevents formation of arthritis in an experimental murine system (35, 36). In addition, enhanced IL-6 levels are observed in the joints and serum of rheumatoid arthritis patients (37). This prompted us to check the expression and secretion of IL-6 in BMDM. We observed expression of IL-6 after 1 h until 72 h of stimulation with PMT (Figure 5A) and the 100-fold increased expression of IL-6 resulted in an enhanced and sustained IL-6 secretion (Figure 5B). These observations suggest that in addition to Tnf-α, IL-6 may help PMT in driving osteoclastogenesis.

We next evaluated the effect of a murine anti-IL-6R antibody (MR-16) on PMT-induced NFATc1, c-Jun, and NF-kB activation. Anti-IL-6R antibody treatment reduced PMT-induced activation of these transcription factors (Figure 5C) and, as a consequence, osteoclast formation (Figure 5D), and expression of osteoclast-specific genes was reduced (Figure 5E). Together these observations suggest that PMT-mediated osteoclast formation relies, at least in part, on the osteoclastogenic effects of IL-6 and Tnf-α.

### PMT Induces Osteoclastogenesis in Absence of IL-1 Receptor

**Figure 6:**
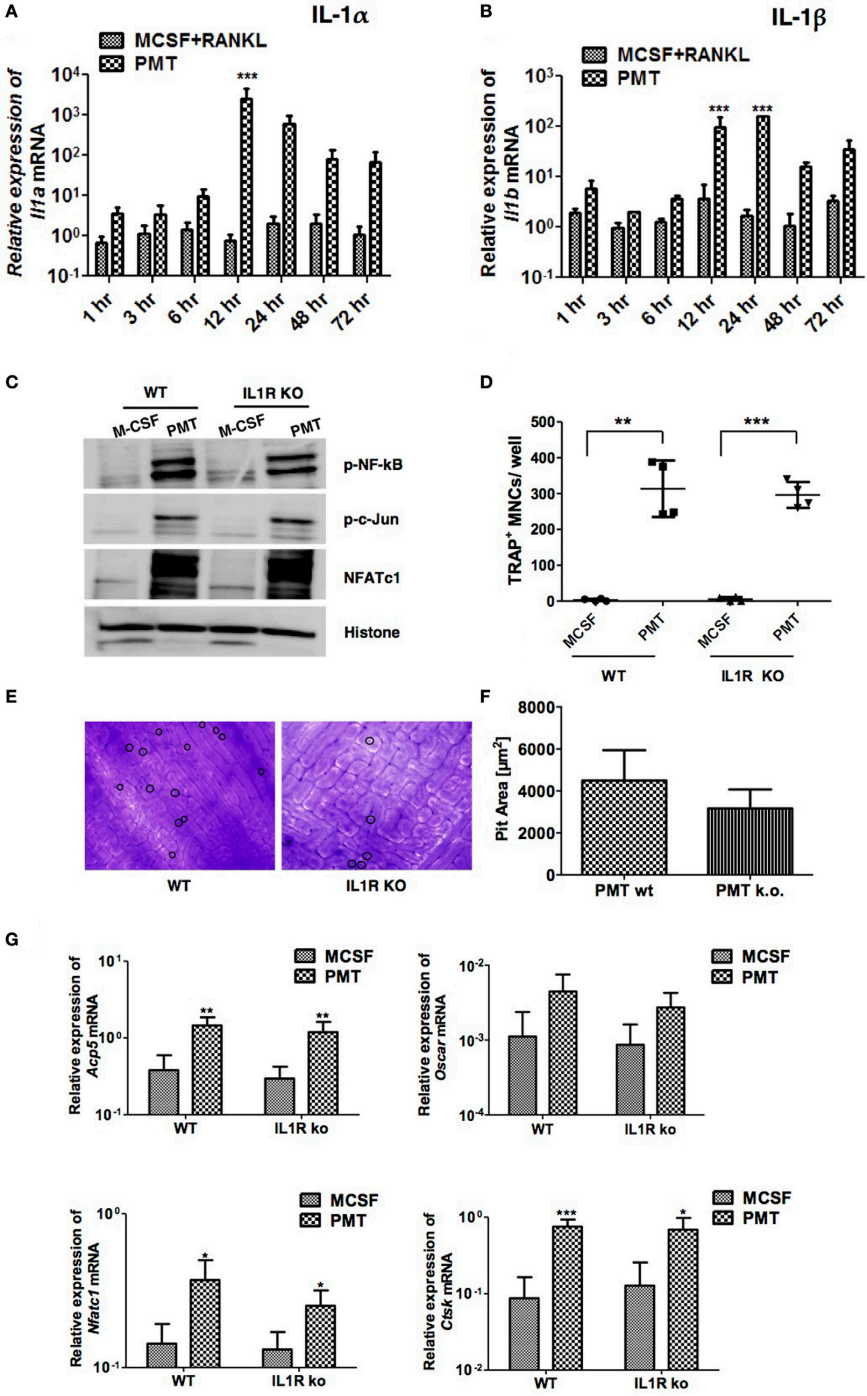
**Absence of interleukin 1 receptor (IL-1R) fails to block *Pasteurella multocida* toxin (PMT)-induced osteoclast formation**. **(A,B)** Bone marrow-derived macrophages (BMDMs) were stimulated as described using standard concentrations of macrophage colony-stimulating factor (M-CSF), M-CSF/sRANKL, or PMT for the indicated time points. Graphs show quantitative RT-PCR analysis of *Il1a* and *Il1b* mRNA levels normalized to *Rps29* expression. The data are presented as fold change relative to the expression of M-CSF-treated cells at the same time point (mean ± SD; *n* ≥ 2). Statistical analysis was done by two-way ANOVA (***p ≤ 0.001). **(C)** BMDMs from wt and IL-1R-deficient mice were stimulated with PMT or M-CSF for 24 h before nuclear extracts were prepared. Immunoblots of phosphorylated NF-κB (p-NF-κB), c-Jun (p-c-Jun), NFATc1, and histone were performed. Histone served as a loading control (*n* = 4). **(D)** BMDMs from wt and IL-1R-deficient mice were stimulated with PMT and M-CSF for 10 days. Multinucleated TRAP^+^ cells were counted; the indicated SD was obtained from four experiments (mean ± SD; *n* ≥ 3). Statistical analysis was performed using a paired Student’s *t*-test comparing TRAP^+^ cells in PMT-treated wells to the M-CSF-treated wells (**p ≤ 0.005; ***p ≤ 0.0005). **(E)** Representative pictures of bone resorption assays using osteoclasts from wt and IL-1R-deficient mice are shown. Resorption pits are marked with a circle. **(F)** Bone resorption assay of BMDMs derived from wt and IL-1R-deficient mice and differentiated into osteoclasts with PMT. The data presented show the pit area that was calculated by subtracting the pit area value of the M-CSF from the PMT-treated condition (*n* = 6). The resorption pit pictures were evaluated in a blinded fashion and false-positive pits were excluded by marking similar structures in M-CSF-treated samples. **(G)** Quantitative RT-PCR analysis of gene expression of *Acp5, Oscar, Nfatc1*, and *Ctsk* in BMDMs derived from wild-type mice or IL-1R-deficient mice, treated with M-CSF or PMT, normalized to *Rps29* expression. Cells were stimulated for 12 h to check the expression of *Nfatc1*; for 24 h to check the expression of *Oscar*; and for 48 h to check the expression of *Acp5* and *Ctsk*. The data are presented as fold change relative to the expression of M-CSF-treated cells at the same time point. The indicated SD was obtained from four experiments (mean ± SD; *n* = 4). Statistical analysis was performed using unpaired Student’s *t*-test comparing gene expression to the M-CSF-treated samples (*p ≤ 0.05; **p ≤ 0.005; ***p ≤ 0.0005).

Like IL-6 and Tnf-α, IL-1α and IL-1β are important osteoclastogenic cytokines (38). Mice deficient in IL-1α, IL-1β, and IL-1α/β suppress arthritis in a mouse model, suggesting that both forms of IL-1 are required for inflammatory bone loss (39). We observed strongly enhanced expression of both, *Il1a* and *Il1b* after 12 h of PMT treatment that lasted until 72 h (Figures 6A,B). As IL-1α and IL-1β transduce signals by binding to IL-1R, we investigated the effect of PMT on BMDM cells derived from IL-1R knockout mice (40). Macrophages deficient in IL-1R showed no differences in the activation of NF-kB, p-c-Jun and NFATc1, respectively (Figure 6C) and IL-1R-deficient BMDM differentiated into TRAP-positive cells similar to wild-type macrophages (Figure 6D). However, when we tested the cells for their ability to resorb bone, IL-1R-deficient osteoclasts treated with PMT seemed to act slightly less efficient, although the difference did not reach statistical significance (Figures 6E,F). Also, we observed comparable expression of osteoclast marker genes after PMT stimulation in IL-1R knockout macrophages (Figure 6G). In summary, these observations suggest that PMT can differentiate macrophages into functional osteoclasts in the absence of IL-1R.

### Cytokines Are Necessary but Not Sufficient for PMT-Induced Osteoclastogenesis

**Figure 7:**
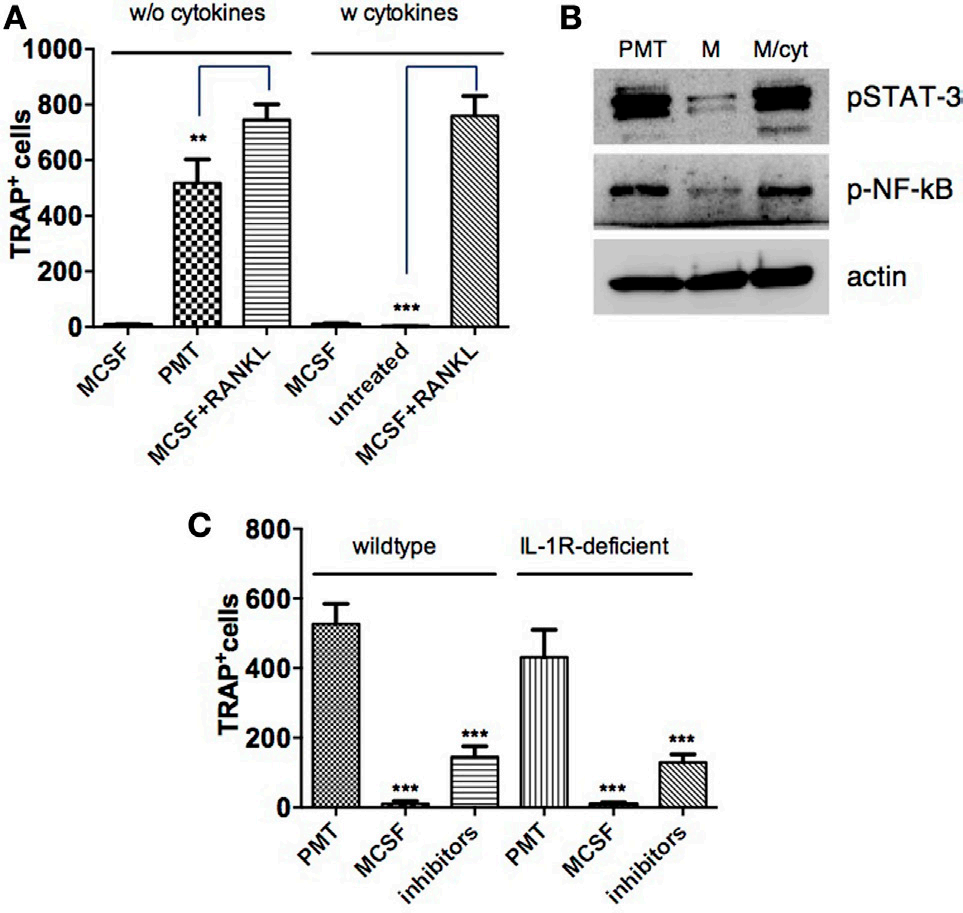
**Cytokines alone are not sufficient to mimic *Pasteurella multocida* toxin (PMT)-stimulated osteoclast differentiation**. **(A)** Bone marrow-derived macrophages (BMDMs) were stimulated with macrophage colony-stimulating factor (M-CSF) or M-CSF/sRANKL with or without addition of cytokines: Tnf-α (700 pg/ml), IL-6 (2,500 pg/ml), and IL-1β (900 pg/ml) and PMT alone for 10–12 days and multinucleated TRAP^+^ cells were counted. The indicated SD was obtained from three experiments (mean ± SD). Statistical analysis was performed using one-way analysis of variance (ANOVA) comparing the difference of tartrate-resistant acid phosphatase (TRAP)-positive cells derived from PMT or cytokine treatment compared to M-CSF/sRANKL-treated control cells. **(B)** BMDMs were stimulated with PMT, M-CSF, or M-CSF with cytokines **(A)** for 1½ days. Total lysates were prepared and immunoblots for phosphorylated STAT-3 (p-STAT-3), phosphorylated NF-κB (p-NF-κB), and β-actin were performed (*n* = 3). **(C)** BMDMs from wt and IL-1R-deficient mice were stimulated with PMT, M-CSF, or PMT in combination with etanercept (120 µg/ml) and the IL-6 neutralizing antibody (105 µg/ml) for 10–12 days. Multinucleated TRAP^+^ cells were quantified and the indicated SD was obtained from three experiments (mean ± SD; *n* = 3). Statistical analysis was performed using one-way ANOVA comparing the decrease of TRAP-positive cells in inhibitor-treated versus PMT-treated control cells.

While it is generally accepted that RANKL is necessary to induce the differentiation of osteoclasts from precursor cells, some publications raise the question whether RANKL-independent osteoclastogenesis can occur under physiological or pathological conditions. It was described that sustained stimulation with cytokines allows osteoclast formation (41, 42). Therefore, we investigated whether the induction of cytokines by PMT is sufficient to induce OC formation. BMDM were treated with the amount of IL-6, Tnf-α, and IL-1β that had been measured in the ELISA experiments. Quantification of TRAP-positive cells showed that the addition of cytokines alone did not induce osteoclast formation, neither in the presence nor absence of a supporting M-CSF stimulus (Figure 7A). In addition, we did not observe any synergistic effect of those cytokines on M-CSF/sRANKL-treated cells. To verify that the cytokine amounts used were appropriate, we checked the activity of specific transcription factors (Figure 7B). Although there was a strong cytokine-specific activation of STAT-3 and NF-κB comparable to that induced by PMT, this activity was insufficient to induce osteoclast formation.

We next studied the effect of an inhibition of all three osteoclastogenic cytokines investigated in osteoclast formation (Figure 7C). Simultaneous inhibition of IL-6 and Tnf-α substantially decreased osteoclast formation by 72% for both WT and IL-1R-deficient mice compared to 63% for etanercept alone and 49% for IL-6R inhibition. These data imply that while cytokines are needed to successfully induce PMT-mediated osteoclast formation, the presence of cytokines alone is not sufficient and that other G-protein-related signaling pathways are required.

## Discussion

*Pasteurella multocida* toxin is a bacterial protein toxin that is known to manipulate host cell signaling cascades which for many cell types result in the activation of mitogenic pathways and the production of cytokines (17). In AR PMT signaling influences bone degeneration by accelerating osteoclast formation and by inhibiting osteoblast differentiation and function. In this study, we show that PMT stimulation of macrophages leads to the induction of osteoclast markers similar to M-CSF/sRANKL stimulation resulting in the differentiation of macrophages into functional osteoclasts. Additionally, we show that PMT-derived osteoclasts are able to resorb bone similar to classical osteoclasts derived from M-CSF/sRANKL stimulation. Strack et al. have recently discussed that PMT-induced differentiation of monocytes into osteoclasts might be RANKL-independent, however, they did not show any experimental proof of their speculation (27). Our data show for the first time that PMT-induced osteoclast formation does not depend on RANKL-RANK signaling. OPG treatment with PMT did not alter the activation of essential transcription factors or the expression of osteoclast marker genes, and the resulting osteoclasts were able to resorb bone without any loss of function.

Additionally, we show for the first time that the PMT-induced inflammatory cytokines such as Tnf-α, IL-6, and IL-1 play a central role in mediating differentiation of macrophages into osteoclasts. Tnf-α alone is a weak inducer of osteoclastogenesis in mouse BMDM (43), but has been reported to induce functional osteoclast formation along with IL-6 (44) or IL-1 (45). Recently, Yarilina et al. have shown that prolonged Tnf-α exposure of human macrophages can trigger differentiation of macrophages into osteoclasts (46). We also observe a sustained production of Tnf-α after PMT stimulation in macrophages, and this continuous exposure of macrophages to Tnf-α may additionally help PMT in inducing osteoclastogenesis. In accordance, our *in vitro* study using etanercept strongly reduced PMT-induced osteoclast formation (Figures 4C–E), suggesting that Tnf-α plays a crucial role in PMT-mediated osteoclastogenesis. PMT stimulation also caused the continuous increased production of IL-6. In rheumatoid arthritis, overproduction of IL-6 is observed and there is a correlation between elevated IL-6 level and clinical indices (47, 48). When we blocked IL-6 signaling, a decrease in PMT-induced osteoclast formation in macrophages was observed (Figures 5C–E). A similar observation was made by Axmann et al. where IL-6R blockade directly suppressed M-CSF/RANKL-induced osteoclast formation (49).

Similar to Tnf-α and IL-6, we observed a high expression of IL-1α and β after PMT stimulation of macrophages. However, our observations in IL-1R knockout mice suggest that PMT can induce differentiation of macrophages into functional osteoclast even in the absence of IL-1R signaling, suggesting that IL-1α and IL-1β do not directly contribute to PMT-induced osteoclast formation in macrophages. However, under physiological conditions, where other IL-1R expressing cells are available, PMT-induced IL-1 production from macrophages may contribute in the progression of AR by either inhibiting osteoblast-mediated bone formation (50) or by inducing the expression of factors supporting osteoclastogenesis such as prostaglandin E2 or RANKL by osteoblasts/stromal cells (51, 52).

The current literature suggests that under physiological conditions the RANKL/RANK axis drives functional osteoclast formation. Pathological conditions, such as cancer or auto-inflammatory bone diseases, are characterized by the presence of a specific microenvironment, where the increased expression of inflammatory cytokines can provide an additional stimulus that further potentiates the generation of bone-resorbing osteoclasts (42, 53). Our data also suggest that the PMT-mediated effect on osteoclasts is probably due to two signaling cascades. While PMT-induced cytokine production seems to be necessary to allow efficient osteoclast formation, additional PMT-mediated signaling is required. Strack et al. recently showed that NFATc1 is activated by PMT downstream of the activated Gαq subunit (27). In addition, NFATc1 overexpression is known to be sufficient for the induction of osteoclastic genes, even in the absence of RANKL (54). Interestingly, NFATc1 expression was not effectively reduced in our inhibitor experiments, and thus, we hypothesize that Gq-dependent expression of NFATc1 could be the additional signal needed for osteoclast formation. This would also explain why inhibition of cytokines did not abrogate osteoclast formation completely, as there is still a robust Gq-mediated induction of NFATc1 transcriptional activity even in the absence of IL-6 and Tnf-α signaling. Collectively, our data suggest that PMT-induced osteoclastogenesis is dependent on cytokines and Gq-mediated signaling but independent of RANKL/RANK axis. A recent article speculates about the possibility of osteoclast subtypes based on large number of studies showing the existence of various subsets of myeloid cells under physiological or pathological state (55). Therefore, we suggest that osteoclasts generated with PMT are distinct from RANKL-derived osteoclasts and are likely to represent a separate subset of osteoclasts; however, detailed future investigations are required for validating our assumption.

Although GPCR signaling is recognized as an important player for many human pathologies, including cardiovascular diseases, inflammation, and cancer, the importance of G proteins in auto-inflammatory bone diseases has not been addressed yet. We recently showed that PMT triggers the differentiation of naïve T cells into a osteoclastogenesis-promoting Th17 phenotype as a consequence of constitutive Gq activation and the resulting downstream activation of STAT-3 (56, 57). This seems to be in contrast with findings of Liu et al. who report that a lack of Gq triggers Th17 differentiation in human lymphocytes (58, 59). However, their data compared mRNA levels but not cellular Gq activity, which for GTPases is a more relevant readout. In support of our findings, other scientists suggest that activation of G proteins by AlF4 treatment activates osteoclast differentiation (60) and that elevated expression of Gq in osteoblasts increases osteoclastogenesis in a transgenic mouse model (61). New therapeutic tools to inhibit G protein signaling are currently being characterized, and it remains to be seen whether they will provide helpful tools in the treatment of auto-inflammatory diseases such as RA as well (62).

## Author Contributions

SC carried out experiments, participated in the design of the study, and drafted the manuscript; BK performed experiments; UH and GS carried out bone resorption studies; and KK generated FACS data, participated in study design, and drafted the manuscript.

## Conflict of Interest Statement

The authors declare that the research was conducted in the absence of any commercial or financial relationships that could be construed as a potential conflict of interest.
