# Supplementary material for: Pasteurella multocida Toxin Triggers RANKL-Independent Osteoclastogenesis
Source: Front Immunol. 2017 Feb 27;8:185. doi: 10.3389/fimmu.2017.00185 (PMC5327351; doi:10.3389/fimmu.2017.00185)
Supplement: Supplementary file 1 [file presentation_1.pdf]

Supplementary Material

Pasteurella multocida Toxin triggers RANKL-independent osteoclastogenesis

S. Chakraborty[1], B. Kloos[1], U. Harre[2], G. Schett[2], and K.F. Kubatzky[1,*]*

Correspondence: Corresponding Author: kubatzky@uni-heidelberg.de

1 Supplementary Figures and Tables

1.1 Supplementary Figure 1

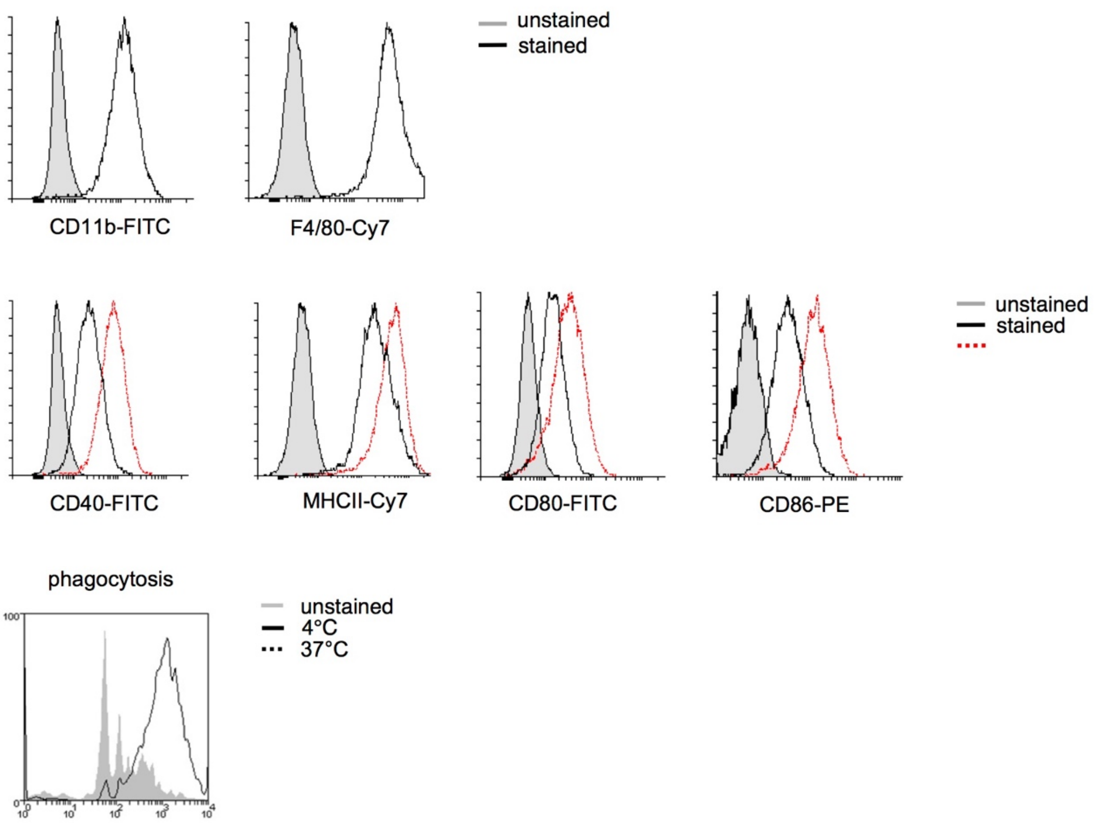

Supplementary Figure 1. Characterization of bone marrow derived macrophage (BMDM) using flow cytometry.

1.2 Supplementary Figure 2

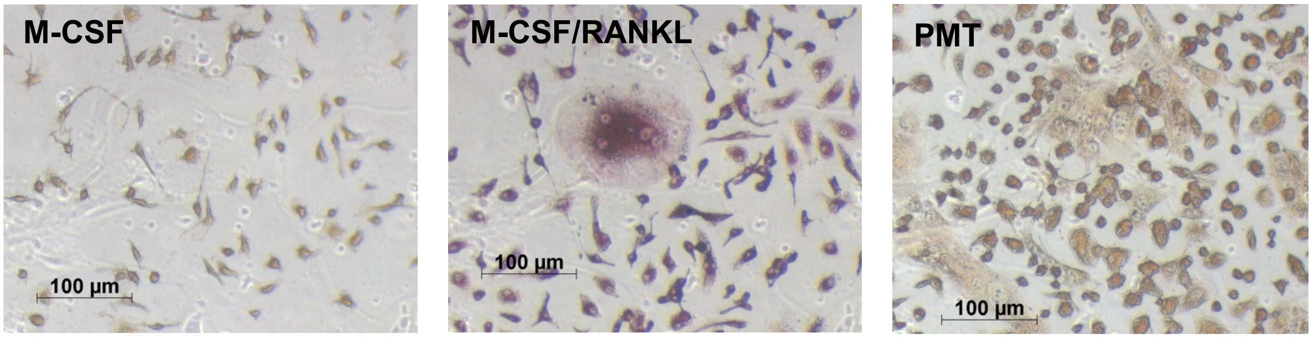

Supplementary Figure 2. BMDMs were stimulated with standard concentrations of M-CSF, M-CSF/sRANKL and PMT for 6-10 days as described in the methods section. Cells were then fixed and stained for TRAP activity. Representative pictures of TRAP-stained osteoclasts formed with PMT and M-CSF/sRANKL are shown (20X magnification).

1.3 Supplementary Figure 3

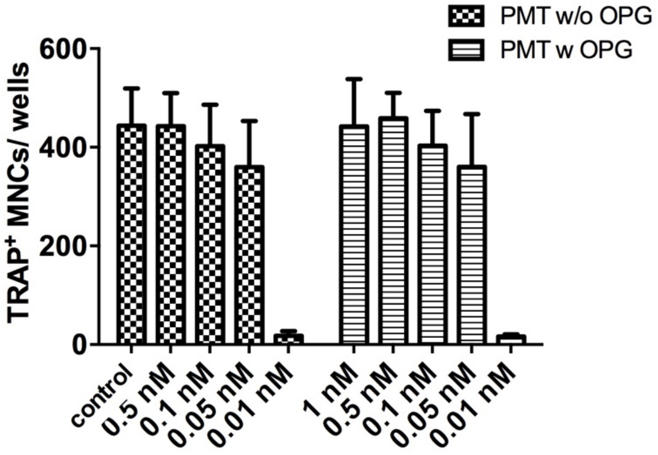

Supplementary Figure 3. BMDMs were stimulated with the indicated concentrations of PMT with or without OPG for 6-10 days. Multinucleated TRAP-positive cells were counted per well. The indicated standard deviation was obtained from three experiments.

1.4 Supplementary Figure 4

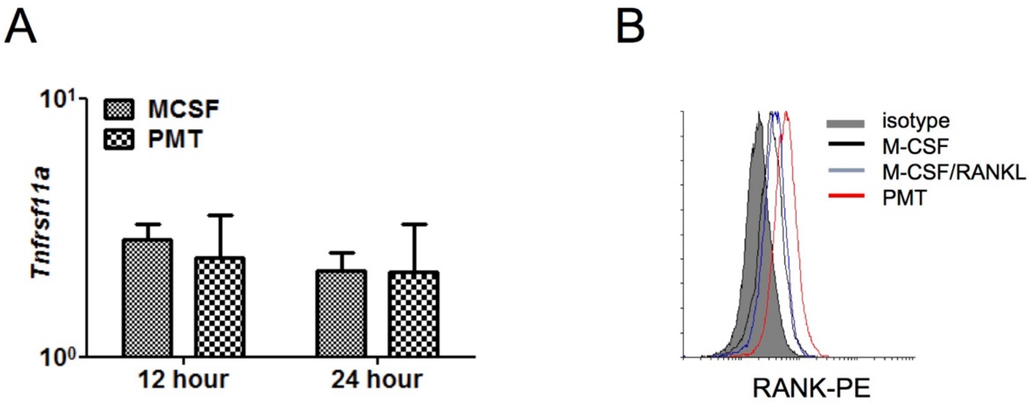

Supplementary Figure 4. Quantitative RT-PCR analysis of *Tnfrsf1 1a*; normalized to *Rps29* expression. Cells were stimulated with PMT (1 nM) or M-CSF (25 ng/ml) for 12-24 hours (mean ± SD; n=3). (B) Representative histogram of RANK surface expression after 24 hours of stimulation with the standard concentrations of PMT, M-CSF and RANKL, respectively.
